# Hypoxia, Snail and incomplete epithelial–mesenchymal transition in breast cancer

**DOI:** 10.1038/sj.bjc.6605369

**Published:** 2009-10-20

**Authors:** K Lundgren, B Nordenskjöld, G Landberg

**Affiliations:** 1 Department of Laboratory Medicine, Center for Molecular Pathology, Malmö University Hospital, Lund University, Malmö SE-205 02, Sweden; 2 Department of Oncology, Borås Hospital, Borås SE-501 82, Sweden; 3 Division of Oncology, Faculty of Health Sciences, Linköping University, Linköping SE-581 85, Sweden; 4 Breakthrough Breast Cancer Research Unit, School of Cancer, Enabling Sciences and Technology, The University of Manchester, Manchester Academic Health Science Centre, Paterson Institute for Cancer Research, The Christie NHS Foundation Trust, Wilmslow Road, Manchester M20 4BX, UK

**Keywords:** hypoxia, EMT, Snail, breast cancer, tamoxifen

## Abstract

**Background:**

Hypoxia is an element of the tumour microenvironment that impacts upon numerous cellular factors linked to clinical aggressiveness in cancer. One such factor, Snail, a master regulator of the epithelial–mesenchymal transition (EMT), has been implicated in key tumour biological processes such as invasion and metastasis. In this study we set out to investigate regulation of EMT in hypoxia, and the importance of Snail in cell migration and clinical outcome in breast cancer.

**Methods:**

Four breast cancer cell lines were exposed to 0.1% oxygen and expression of EMT markers was monitored. The migratory ability was analysed following Snail overexpression and silencing. Snail expression was assessed in 500 tumour samples from premenopausal breast cancer patients, randomised to either 2 years of tamoxifen or no adjuvant treatment.

**Results:**

Exposure to 0.1% oxygen resulted in elevated levels of Snail protein, along with changes in vimentin and E-cadherin expression, and in addition increased migration of MDA-MB-468 cells. Overexpression of Snail increased the motility of MCF-7, T-47D and MDA-MB-231 cells, whereas silencing of the protein resulted in decreased migratory propensity of MCF-7, MDA-MB-468 and MDA-MB-231 cells. Moreover, nuclear Snail expression was associated with tumours of higher grade and proliferation rate, but not with disease recurrence. Interestingly, Snail negativity was associated with impaired tamoxifen response (*P*=0.048).

**Conclusions:**

Our results demonstrate that hypoxia induces Snail expression but generally not a migratory phenotype, suggesting that hypoxic cells are only partially pushed towards EMT. Furthermore, our study supports the link between Snail and clinically relevant features and treatment response.

Epithelial–mesenchymal transition (EMT) is a biological process defining progression from a polarised epithelial phenotype to a mesenchymal phenotype, which is distinguished by fibroblast-like features (Thiery and Sleeman, 2006). Epithelial–mesenchymal transition is characterised by a change in the cell-type-specific protein repertoire and facilitation of cell motility, is essential for generation of new tissue types during embryogenesis, and has a pivotal role in inflammation and wound healing of adult tissue (Thiery, 2003; Flint *et al*, 2008). In addition, it has been implicated in the fundamental steps of tumorigenesis such as invasion and metastasis (Sleeman, 2000). Epithelial–mesenchymal transition is considered a transient and reversible process and represents only one of several steps required for tumour progression via invasion and metastatic spread (Christiansen and Rajasekaran, 2006). As reviewed by Christiansen *et al* there are several reports describing a partial or incomplete EMT phenotype of advanced carcinomas, displaying some mesenchymal features, but with a retention of well-differentiated epithelial-cell characteristics (Christiansen and Rajasekaran, 2006).

The architectural arrangement of an epithelial tissue is characterised by an intricate network of junctional structures such as tight, gap and adherens junctions and desmosomes. Cadherins are important for stabilisation of these structures, and in particular E-cadherin (Matter and Balda, 2003). An important hallmark of EMT is loss of E-cadherin expression, resulting in destabilisation of the cell–cell contacts and detachment of cells from its surroundings. This crucial event results from repression of the E-cadherin gene transcription via overexpression of several different EMT-inducing repressors, such as the zinc-finger transcription factors Slug (Hajra *et al*, 2002) and the focus of our study; Snail (Barrallo-Gimeno and Nieto, 2005).

Overexpression of Snail has been reported to be a sufficient inducer of EMT as well as being predictor for an aggressive tumour phenotype (Cano *et al*, 2000). In breast cancer increased Snail expression has been associated with lymph node involvement, invasiveness and metastatic potential (Cheng *et al*, 2001; Blanco *et al*, 2002), and also with decreased recurrence-free survival (Moody *et al*, 2005). Kurrey *et al* (2005) reported ectopic Snail or Slug expression to result in induction of EMT in an ovarian carcinoma cell line, with enhanced motility and invasiveness, loss of expression of epithelial markers and increased expression of vimentin. Stable silencing of endogenous Snail concomitantly induced complete mesenchymal–epithelial transition in MDCK-Snail cells, and reduction of *in vivo* tumour growth of two independent, stably Snail-silenced carcinoma cell lines (Olmeda *et al*, 2007).

Besides the importance of the tumour cell itself, it is well established that the tumour microenvironment also plays a crucial role in carcinogenesis (Liotta and Kohn, 2001). Hypoxia is a common feature of solid tumours and affects several important biological processes, and is also linked to clinical aggressiveness (Harris, 2002). Hypoxia is predominantly caused by abnormal vasculature formation of the rapidly growing tumour mass, and the net result is heterogeneously distributed areas of low oxygen pressure (Vaupel *et al*, 2002). Tumour cell adaptation allows survival and gives rise to heterogeneity and selection of resistant clones, consequently resulting in a more malignant phenotype (Semenza, 2003). The hypoxic response mediated by the transcription factor hypoxia-inducible factor-1 (HIF-1) activates target genes involved in a number of different cellular processes, such as proliferation, angiogenesis and EMT, all linked to malignant progression (Semenza, 2002; Imai *et al*, 2003; Yang and Wu, 2008). Upregulation of Snail mRNA expression under hypoxic conditions, accompanied by downregulation of E-cadherin, has been reported in ovarian cancer cell lines (Imai *et al*, 2003; Kurrey *et al*, 2005), and invasiveness of hypoxic ovarian cancer cell lines has also been shown to increase (Imai *et al*, 2003). Furthermore, Lester *et al* (2007) reported that exposure of the human breast cancer cell line MDA-MB-468 to 1% oxygen resulted in increased vimentin expression, decreased E-cadherin levels and nuclear translocation of the Snail protein. Notably, these cells adopted a mesenchymal-like morphology, increased their migratory capacity and were able to disseminate *in vivo*.

Becker *et al* (2007) published a summary of 21 studies, where 10 were conducted using breast carcinomas, describing the expression of Snail with regard to clinicopathological parameters and disease outcome in a large number of samples from several different cancers. This report points out the discrepancy between studies and the use of different Snail antibodies, and emphasises that selection of novel Snail-specific antibodies may facilitate the establishment of a potential clinical importance for this EMT regulator.

In this study we, thus, focused on elucidating the regulation of EMT via Snail induction, and on the relationship between EMT and hypoxia, in a panel of breast cancer cell lines, using a well validated Snail-specific antibody. Furthermore, the importance of Snail expression in primary breast cancer was examined in a clinical material consisting of 500 invasive premenopausal breast cancer cases, and in full sections of ductal carcinoma *in situ* (DCIS). We show that Snail protein expression increased in breast cancer cell lines exposed to severe hypoxia (0.1%) and that expression of E-cadherin and the mesenchymal marker vimentin were also affected in some cell lines. Nuclear localisation of the protein further seemed to be linked to the EMT-inducing function of Snail. Cell motility increased in one cell line only, following exposure to hypoxia, which turned out to be independent of Snail. Moreover, we demonstrate that Snail expression is important for migration of breast cancer cell lines, through overexpression and silencing of the protein. Finally, nuclear expression of Snail was shown to be associated with higher grade and high proliferation rate in primary breast cancer. In addition, we for the first time show that Snail is a predictor for tamoxifen response.

## Materials and methods

### Cell lines, Western blot and immunocytochemistry

For this study we used the human breast cancer cell lines MCF-7, T-47D, MDA-MB-468 and MDA-MB-231 (ATCC, Manassas, VA, USA). MCF-7 cells were grown in Improved MEM zinc option supplemented with 5% fetal bovine serum (FBS) and 1% penicillin/streptomycin (PEST), T-47D in DMEM supplemented with 10% FBS and 1% PEST and MDA-MB-468 and MDA-MB-231 in RPMI 1640 supplemented with 10% FBS, 1 mM sodium pyruvate and 1% PEST. Cell lysates and pellets were made from the cell lines exposed to 0.1% O_2_ for increasing time points from 2 to 96 h, where time point zero defined normoxia (21% O_2_). Cells were seeded to avoid confluence at the time for harvest. For immunocytochemistry (ICC) cells were harvested by trypsinisation, washed in PBS, fixed in 4% paraformaldehyde for 25 min, stained with Meyer's haematoxylin for 5 min and centrifuged at 1500 r.p.m. for 5 min with the subsequent pellet resuspended in ice-cold 70% ethanol. Pellets were dehydrated in graded ethanol series, embedded in paraffin and a cell-line array was constructed and stained with antibodies, according to staining procedures described below. For the ICC staining of HIF-1*α* the number of HIF-1*α*-positive cells was counted in three equal fields for each time point and cell line, with an average calculated for each time point.

For Western blot analysis cells were trypsinised, centrifuged, washed and pellets were stored at −80°C for 1 h. Cells were then lysed in buffer containing 0.5% NP-40, 0.5% NaDOC, 0.1% SDS, 50 mM TRIS (pH 7.0), 150 mM NaCl, 1 mM EDTA (pH 8.0), 1 mM NaF, 0.1 mg/ml PMSF and a Complete Mini protease inhibitor cocktail tablet (Roche, Mannheim, Germany). Cell extracts were incubated on ice for 30 min, with vortexing every 10 min, and centrifuged at 14 000 r.p.m for 30 min. Supernatants were collected and protein concentrations were measured with a BCA Protein Assay kit (Pierce, Rockford, IL, USA). For each protein sample 10 *μ*g was resolved on SDS–polyacrylamide gels and then transferred to Hybond ECL nitrocellulose membranes (Amersham Pharmacia Biotech, Buckinghamshire, UK). Membranes were incubated with rat monoclonal anti-human Snail (1:400, clone SN9H2; Cell Signaling Technology, Beverly, MA, USA), mouse monoclonal anti-human E-cadherin (1:500, clone 4A2C7; Zymed Laboratories, Invitrogen immunodetection, San Francisco, CA, USA), mouse monoclonal anti-human vimentin (1:250, clone V9; Dako, Glostrup, Denmark) antibody and polyclonal goat anti-human *β*-actin antibody (1:500; Santa Cruz Biotechnology, Santa Cruz, CA, USA) for 2 h followed by incubation with secondary horseradish peroxidase-conjugated anti-rat or mouse (Amersham Life Science, Alesbury, UK) and anti-goat antibodies (Sigma, Gothenburg, Sweden) for 1 h. Membrane-bound antibody was detected by using the ECL^+^ system (Amersham Life Science, Alesbury, UK).

### Transfections

For transient expression of wild-type Snail we used the HA-Snail-246A plasmid (Yang *et al*, 2005) provided by Dr Kumar (MD Andersen Cancer Center, Houston). For transfections of MCF-7 and T-47D in six-well plates the Amaxa Nucleofector kit V was used according to the manufacturer's protocol (Amaxa Inc., Gaithersburg, MD, USA). A 2-*μ*g weight of DNA was added per well of a six-well plate. MDA-MB-468 and MDA-MB-231 were transiently transfected with Snail vector using Lipofectamine 2000 according to the manufacturer's recommendations (Invitrogen Life Technologies, Carlsbad, CA, USA). A 2-*μ*g weight of DNA was used per well of a six-well plate. For Snail knockdown MDA-MB-468 and MDA-MB-231 cells were transfected with 50 nM control siRNA or siRNA against Snail (ON-TARGET *plus* siRNA SMARTpool; Dharmacon, Lafayette, CO, USA) using Dharmafect (Dharmacon, Lafayette, CO, USA). Cells were allowed to grow 24 or 48 h after transfection before harvesting for migration assay.

### Migration assay

Migration assays were performed using Transwell migration chambers with 8 *μ*m pore membranes (Corning Inc., Corning, NY, USA). Cells (10^5^) were resuspended in upper Transwell chambers in serum-free media and allowed to migrate towards a serum gradient (10%) in the lower chamber for 3 h (MDA-MB-231) or 6 h (MCF-7, T-47D and MDA-MB-468). Membranes were then excised and cells on the upper side were removed before fixing in 4% paraformaldehyde and staining with DAPI (4′6-diamidino-2-phenylindole), prior to mounting. The number of migrating cells was counted in three randomly chosen fields on each membrane and photographed at × 10 magnification. Values reported are the averages of three experiments performed in triplicates.

### Patient material

#### Patient cohort

Between 1986 and 1991, 564 premenopausal breast cancer patients with invasive stage-II disease were enrolled in a Swedish trial (SBII:2a) where they were randomly assigned to 2 years of adjuvant tamoxifen (*n*=276) or no adjuvant treatment (control, *n*=288). The aim of the original study was examine the effect of tamoxifen treatment (20 or 40 mg daily) on recurrence-free survival. Patients were included irrespective of hormone receptor status. All patients were followed up for recurrence-free and overall survival. Recurrence was defined as local, regional or distant recurrence, and breast cancer-specific death. A new contralateral breast cancer was not recorded as recurrence, but was separately analysed. Surgery was modified radical mastectomy or breast conserving surgery, followed by radiotherapy, and in few cases adjuvant polychemotherapy (less than 2%). The median post-surgery follow-up time without breast cancer event was 13.9 years. Detailed description of the SBII:2a study can be further obtained from a previous report (Ryden *et al*, 2005). Informed consent was obtained from the patients participating in the randomised study and the ethical committees at Lund and Linköping Universities approved the study and the reanalysis of the tumour material.

### Ductal carcinoma *in situ*

Twenty-five full sections taken from patients with DCIS were chosen from archived formalin-fixed, paraffin-embedded surgical breast tumour specimens at Department of Pathology, Malmö University Hospital. Inclusion criteria were symmetrical DCIS lesions with central necrosis.

### Tissue specimens and immunohistochemistry

Formalin-fixed, paraffin-embedded tumour material was available from 500 of the 564 patients in the trial. Areas representative of invasive cancer were selected and assembled in a tissue microarray (TMA). Two 0.6-mm tissue cores from each donor block were placed in recipient paraffin blocks using an automated tissue arrayer (Beecher Instruments Microarray Technology, Woodland, MD, USA). Sections (4 *μ*m) from this block were mounted on slides before they were deparaffinised, rehydrated and microwave-treated in target retrieval solution at pH 9.9 (Dako, Glostrup, Denmark), before being processed with an automated immunostainer (Techmate 500; Dako, Copenhagen, Denmark), using the Envision software (Dako, Glostrup, Denmark). The antibody employed was Snail (1:100). Staining was evaluated by two independent observers (one pathologist), to obtain a result as correct and representative as possible. Conflicting observations were low (<5%) for all evaluations made. All immunohistochemical (IHC) evaluations were performed without knowledge of tumour characteristics. In cases of no evaluation, cores were either non-representative (i.e., contained no invasive tumour cells) or missing. Data for expression of the oestrogen receptor-*α* (ER*α*) and the progesterone receptor (PR) were available from a previous study (Ryden *et al*, 2005). Expression of the proliferation marker Ki67 had also been evaluated in a previous study (Jirstrom *et al*, 2005). Carbonic anhydrase-IX (CAIX) staining (Brennan *et al*, 2006) as well as HIF-1*α* staining (Kronblad *et al*, 2006) had also previously been evaluated. Formalin-fixed, paraffin-embedded full section DCIS and arrays of cell pellets were treated as previously described. Antibodies employed were Snail (1:100), E-cadherin (1:100), vimentin (1:1000) and mouse monoclonal anti-human HIF-1*α* (1:100, clone 54; BD Biosciences, Erembodegem, Belgium).

### ICC evaluation of cytoplasmic and nuclear Snail expression

SlidePath's Positive Pixels Algorithm (Digital Slide Server) was used for evaluation of ICC staining of cytoplasmic Snail protein in the different cell lines. The algorithm detects positively stained pixels within the image. The analysis was performed on three randomly chosen fields for each time point and cell line, on the array made from cell pellets, and an average was calculated. For more detailed description we refer to the Tissue Image Analysis User Guide (SlidePath Ltd., Dublin, Ireland). For the ICC staining of nuclear Snail the percentage positive nuclei was manually counted in three equal fields for each time point and cell line, with an average calculated for each time point.

### Statistical methods

Statistical analyses were performed using the SPSS software (version 15.0; SPSS, Chicago, IL, USA). The Wilcoxon/Mann–Whitney test and Fisher's exact test were used for associations between Snail expression and clinicopathological parameters and tumour markers. To study recurrence-free survival the Kaplan–Meier method was used, and the log-rank test was applied for comparison of recurrence-free survival among different treatment groups. A Cox proportional-hazards regression model was used for the estimation of relative risk in multivariate analysis. All *P*-values corresponded to two-sided tests and a *P*-value less than 0.05 was considered statistically significant.

## Results

### Snail is expressed in breast cancer cell lines

Prior to this study we had been evaluating a number of different Snail antibodies and found that some were unspecific and reacted also with the closely related family member Slug. The reactivity of the Snail-specific antibody used in our study was further validated through comparison of the expression pattern by Western blot and immunocytochemical analysis of protein lysates and pellets made from the human breast cancer cell lines MCF-7, T-47D, MDA-MB-468 and MDA-MB-231. Both methods revealed a similar expression pattern for the protein in the cell lines (data not shown). Staining was both nuclear and cytoplasmic, as observed by ICC. In Western blot a band corresponding to the predicted molecular weight for Snail was obtained at 29 kDa (Supplementary Figure 1). Bands corresponding to the EMT markers E-cadherin and vimentin were obtained at ∼100 and 54 kDa, respectively (Supplementary Figure 1).

### Hypoxia induces an EMT phenotype in breast cancer cell lines

**Figure 1:**
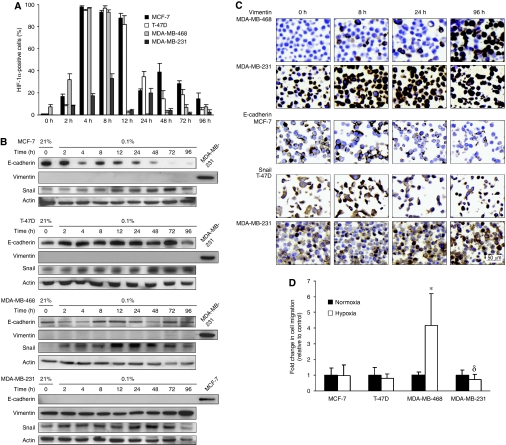
EMT marker expression and migratory capacity in breast cancer cell lines exposed to hypoxia. (**A**) HIF-1*α* expression representing the hypoxic response peaked between 4 and 8 h in all cell lines, and again at 48 h but with a lower expression level in MCF-7 cells, and at 24 h in MDA-MB-231 cells. (**B**) Snail expression increased in all the cell lines, with a slight variation in expression levels over time. E-cadherin protein levels decreased in MCF-7 cells starting at 4 h and was significantly reduced after 72 h, and fluctuated over time in MDA-MB-468 cells. A slight increase of vimentin expression was detected in Western blot in MDA-MB-468 and MDA-MB-231 cells. (**C**) Vimentin expression detected by ICC increased in MDA-MB-468 and MDA-MB-231 cells upon hypoxic exposure, and was markedly upregulated at 96 h in both cell lines. The ICC confirms downregulation of E-cadherin in MCF-7 cells and increased levels of both cytoplasmic and nuclear Snail is exemplified for T-47D and MDA-MB-231 cells. (**D**) The migratory ability of MDA-MB-468 cells increased three-fold (^*^P<0.001) *vs* control when cells were exposed to 0.1% oxygen for 48 h, whereas significantly decreased in hypoxic MDA-MB-231 cells (^*δ*^*P*<0.001) *vs* control. The result in panel **D** is presented as means of three experiments.

First, the effect of hypoxia on EMT regulation was studied in the four cell lines. Cells were exposed to 0.1% oxygen from 2 to 96 h. Expression of HIF-1*α* was used to visualise the hypoxic response, and the expression pattern differed between the four cell lines. Expression of HIF-1*α* peaked between 4 and 8 h in all cell lines, and then declined (Figure 1A). For MCF-7, and MDA-MB-231 a less prominent expression peak was observed at 48 and 24 h respectively. Interestingly, exposure to 0.1% oxygen increased the protein levels of Snail in all cell lines. Snail expression increased over time, but with a slightly different expression pattern among cell lines, as demonstrated by Western blot (Figure 1B). Similar observations were made on ICC stainings of cell pellets, as shown for T-47D and MDA-MB-231 (Figure 1C). Cytoplasmic intensity as well as number of cells exhibiting nuclear Snail expression increased over time. E-cadherin protein expression, which was present in all cell lines except for MDA-MB-231, decreased only in MCF-7 cells, starting at approximately 4 h, and after 72 h the expression was significantly reduced (Figure 1B and C). Expression of the mesenchymal marker vimentin, which is expressed only by MDA-MB-231 cells at normoxia, increased during exposure of MDA-MB-468 and MDA-MB-231 to hypoxia, more easily detectable by ICC (Figure 1B and C). Proliferation, as measured by Ki67, was maintained at a high level throughout the time course of hypoxic exposure (Supplementary Figure 2). Since EMT is associated with tumour invasiveness and metastasis, we wanted to confirm that EMT-induced cells are motile. Interestingly, we found that the migratory capacity of MDA-MB-468 cells exposed to hypoxia for 48 h increased three-fold relative to normoxic cells (Figure 1D). However, migration was not affected in MCF-7 and T-47D cells, and in contrast, MDA-MB-231 cells exhibited significantly lower migratory ability when exposed to hypoxia (Figure 1D). Taken together, exposure of these breast cancer cell lines to hypoxia induced partial EMT that affected the migratory ability in one of the four cell lines (Table 1).

### Subcellular localisation of Snail is affected by hypoxia

**Figure 2:**
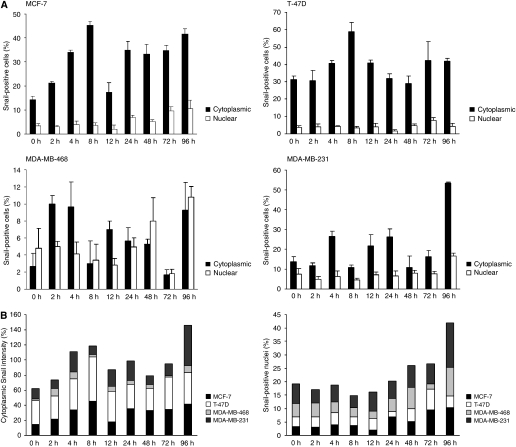
Subcellular localisation of Snail in breast cancer cells exposed to hypoxia. (**A**) In MCF-7 cells hypoxia induced cytoplasmic Snail expression with a peak at 8 h, followed by a decline at 12 h and again an increased expression. Nuclear expression was increasing over time and peaked at 96 h. Expression of Snail in T-47D fluctuated over time, with a cytoplasmic expression peak at 8 h, but no prominent expression peak in the nuclear expression was observed. Cytoplasmic Snail expression fluctuated over time in MDA-MB-468, with somewhat lower levels between 8 and 72 h, whereas nuclear expression peaked at 96 h. In MDA-MB-231 cells cytoplasmic as well as nuclear expression showed a prominent peak at 96 h. (**B**) When adding expression levels of all cell lines, two different expression peaks were observed for cytoplasmic expression, the first one at 8 h and the second at 96 h. Nuclear Snail expression reached its highest levels at 96 h, when presented for all the cell lines.

Our next objective was to analyse the subcellular localisation of Snail as an effect of hypoxic exposure. Cytoplasmic staining intensity, measured by the Positive Pixels Algorithm (developed by SlidePath Ltd., Dublin, Ireland), increased over time. In all four cell lines the expression levels peaked between 2 and 8 h, and then again between 72 and 96 h (Figure 2A). The lowest expression of cytoplasmic Snail was observed in MDA-MB-468 cells. In contrast to the cytoplasmic expression, nuclear expression started to increase later during hypoxic exposure and peaked at 96 h in all cell lines except for in T-47D. Nuclear expression in T-47D was retained at a quite low level throughout the time course of exposure (Figure 2A). Interestingly, the elevated levels of nuclear Snail detected at later time points corresponded to those in time points when prominent changes in E-cadherin in MCF-7 cells, and vimentin expression in MDA-MB-468 and MDA-231 were observed. Adding the expression levels of all the cell lines, two expression peaks for cytoplasmic expression were observed, one at 8 h and the second at 96 h (Figure 2B). Since nuclear expression peaked at 96 h in all cell lines except for T-47D, one prominent peak was detected at 96 h when adding the expression of all the cell lines (Figure 2B).

### Overexpression and silencing of Snail impacts upon breast cancer cell migration

**Figure 3:**
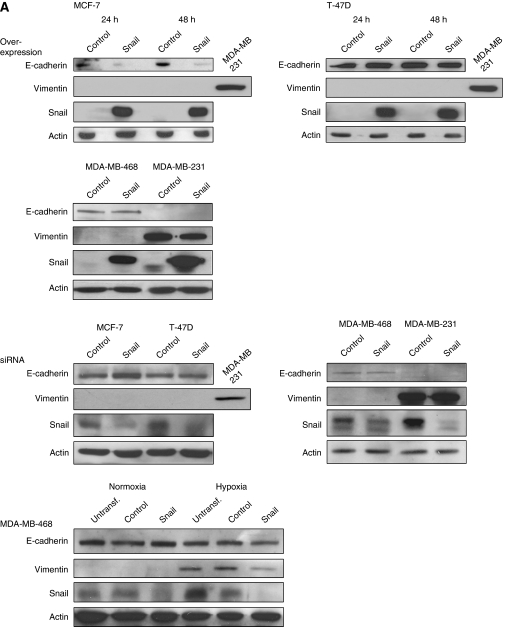

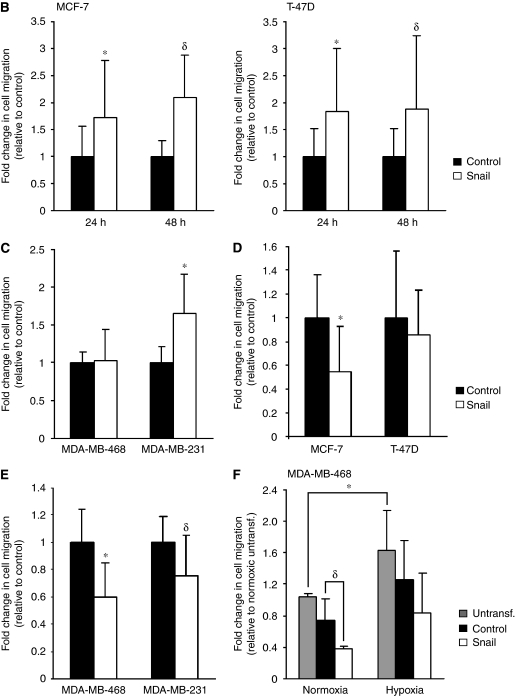
Effect of transient overexpression and silencing of Snail on the migratory ability of breast cancer cell lines. (**A**) Western blots confirming overexpression and knockdown of Snail and expression of E-cadherin and vimentin for the four cell lines. In MCF-7 cells transfected with Snail vector a decrease in E-cadherin protein levels was observed, and a slight increase in E-cadherin expression could be detected when Snail was silenced. Absence of bands in the control lanes for overexpression was due to short exposure times. In MDA-MB-468 cells exposed to hypoxia, vimentin expression decreased following Snail knockdown. (**B**) Snail overexpression increased migration of MCF-7 and T-47D cells 24 h (^*^, MCF-7: *P*=0.034; T-47D: *P*=0.025) and 48 h (*δ*, MCF-7: *P*=0.002; T-47D: *P*=0.043) post transfection. (**C**) The migratory ability of Snail-overexpressing cells increased also in MDA-MB-231 cells (^*^P<0.001) *vs* control 48 h post transfection, whereas no change was observed in MDA-MB-468 cells. (**D**) Knockdown of Snail resulted in decreased migration of MCF-7 cells (^*^P<0.001) *vs* control, but no effect was observed in T-47D cells (**E**) Snail silencing in MDA-MB-468 and MDA-MB-231 decreased the migratory ability *vs* control 48 h post transfection (^*^P<0.001, *^δ^P*<0.001). (**F**) Snail knockdown in MDA-MB-468 cells exposed to hypoxia did not exhibit the decreased migratory propensity observed for normoxic cells treated with siRNA against Snail. There was a significant difference in migration between normoxic and hypoxic cells (^*^P=0.028), and between normoxic control-transfected and Snail-transfected cells (*^δ^P*=0.023). The results are presented as means of three experiments. Wide error bars for MCF-7 and T-47D cell migrations are due to low and varying cell number between experiments.

In order to further investigate the role of Snail in cell motility, we transiently overexpressed and silenced the protein in the cell lines (Figure 3A). With the exception of MDA-MB-468 cells, the moderate increase in Snail expression following hypoxic exposure did not increase the migratory capacity of the cell lines. Hence, we wanted to elucidate the effect of a prominent increase in Snail expression on migration. The migratory capacity of the epithelial-like cell lines MCF-7 and T-47D is low, MDA-MB-468 moderate, and the mesenchymal-like MDA-MB-231 is highly migratory (Supplementary Figure 3). Overexpression of Snail in MCF-7 and T-47D cells resulted in significant increase in cell motility, 24 and 48 h post transfection, relative to the control (Figure 3B). Notably, migration of T-47D was effected even though E-cadherin was not downregulated as a result of elevated Snail levels (Figure 3A). Furthermore, Snail overexpression resulted in increased migration of MDA-MB-231 cells, whereas no effect on MDA-MB-468 cell migration was observed (Figure 3C). No significant changes in the expression of either E-cadherin or vimentin were found when Snail was overexpressed in these two cell lines (Figure 3A). To exclude the possibility that increased migration following overexpression of Snail was just an artefact of the actual overexpression, we, in addition, overexpressed the closely related E-cadherin repressor Slug in the cell lines. This however, did not result in enhanced migratory capacity in any of the cell lines (data not shown). Moreover, silencing of Snail expression in MCF-7, MDA-MB-468 and MDA-MB-231 decreased cell migration significantly, whereas no effect of the knockdown was observed in T-47D cells (Figure 3D and E). Notably, E-cadherin and vimentin levels were unaffected when Snail was knocked down, except for a slightly elevated E-cadherin level in MCF-7 cells (Figure 3A).

We further wanted to delineate the link between hypoxia induced Snail expression and migration in MDA-MB-468 cells, since the migratory capacity of this only cell line increased as a result of exposure to hypoxia. Snail was silenced by siRNA and cells were grown for 48 h in either normoxia (21% oxygen) or in hypoxia prior to migration. We again observed a significantly decreased migration of normoxic cells transfected with siRNA against Snail, relative to the control (Figure 3F). However, motility of hypoxic cells transfected with siRNA against Snail was in contrast not significantly decreased due to the knockdown, relative to control-transfected hypoxic cells (Figure 3F). These results indicate that the increased migratory ability of MDA-MB-468 cells observed in hypoxia was independent of Snail, and may instead be dependent on another factor, or a number of different factors activated by the hypoxic response.

### Nuclear Snail expression is associated with higher grade and proliferation rate, and is a predictive marker for tamoxifen response in premenopausal breast cancer

**Figure 4:**
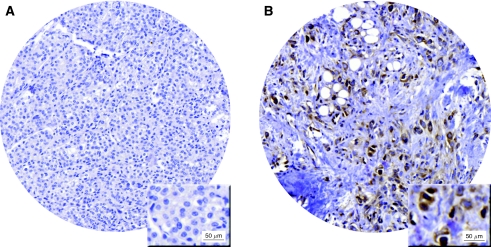
Expression of Snail in primary breast tumours. (**A**) Tumour core exhibiting absence of Snail expression. (**B**) A fraction of tumours showed presence of nuclear Snail expression.

**Figure 5:**
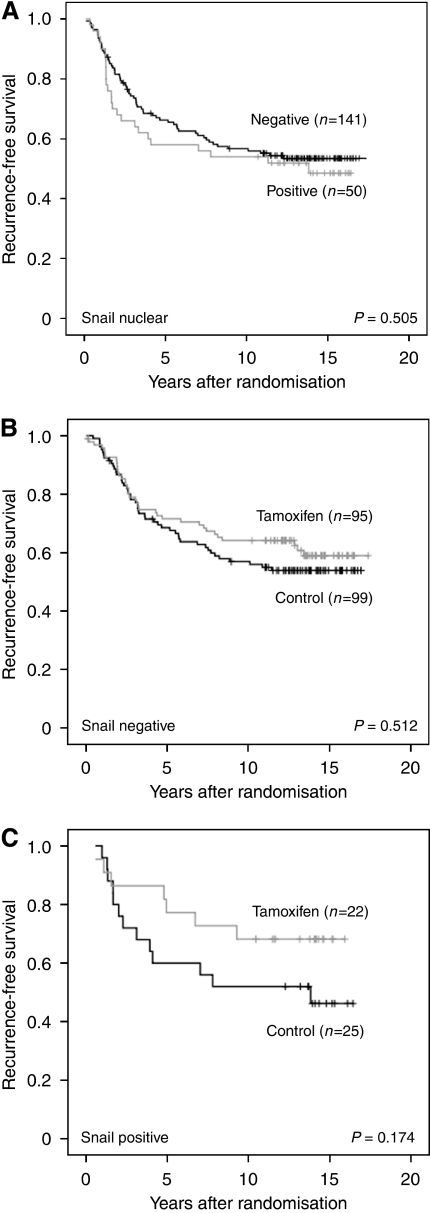
Kaplan–Meier curves showing the effect of Snail on recurrence-free survival. (**A**) In the subgroup of untreated control patients expression of nuclear Snail had no effect on the recurrence rate. (**B**) For patients with ER*α*-positive, Snail-negative tumours no difference in recurrence-free survival was observed between untreated and treated patients. (**C**) A trend towards shorter recurrence-free survival for untreated ER*α*-positive, Snail-positive patients as compared with treated patients, was observed.

Next, we examined potential associations between Snail expression and tumour characteristics. The tumour material collected from a clinical trial of patients randomised to either 2 years of tamoxifen or no adjuvant treatment after surgery allowed us to investigate the prognostic effect of Snail expression. Nuclear staining was assessed as being absent (0) (Figure 4A) or present (1) (Figure 4B). Based on those criteria 293 (76.3%) and 91 (23.7%) tumours out of the 384 that were assessable, displayed absence or presence of nuclear Snail staining respectively (Table 2). The majority of tumours displayed cytoplasmic staining of varying intensity, but we chose to focus on nuclear staining since Snail is active in the nucleus, with its functional role as a transcriptional repressor of the E-cadherin gene. Interestingly, expression of Snail was positively correlated to tumour grade (*P*=0.003) and to expression of the proliferation marker Ki67 (*P*=0.024), implying that nuclear Snail expression is associated with markers defining a more aggressive disease in breast cancer (Table 2). However, no associations with clinicopathological parameters such as tumour size, tumour type or lymph node status were found. Moreover, nuclear expression of Snail was inversely correlated to expression of both the ER*α* (*P*=0.002) and the PR (*P*=0.006) (Table 2). No correlation was observed between Snail expression and HIF-1*α*, but a positive correlation to expression of the hypoxia marker CAIX (*P*=0.040) was found, suggesting a possible link between EMT and hypoxia in breast cancer (Table 2). Notably, cytoplasmic Snail expression was not associated with any of the variables described. We examined recurrence-free survival among patients in the untreated control group according to Snail expression; however, Snail expression had no prognostic value (Figure 5A). Survival analyses revealed a trend towards impaired tamoxifen response among patients with Snail-negative tumours, compared with the those having Snail-positive tumours (Figure 5B and C). Interestingly, when analysing patients randomised to tamoxifen treatment *vs* no adjuvant treatment, using a multivariate Cox proportional-hazards regression model for Snail and treatment interaction, a statistically significant difference between the two subgroups defined by nuclear Snail expression was observed (*P*=0.048) (Table 3). These results indicate that patients with tumours exhibiting no nuclear Snail expression are less likely to respond to tamoxifen, as compared with patients with Snail-positive tumours.

### Hypoxic cells of DCIS express nuclear Snail

**Figure 6:**
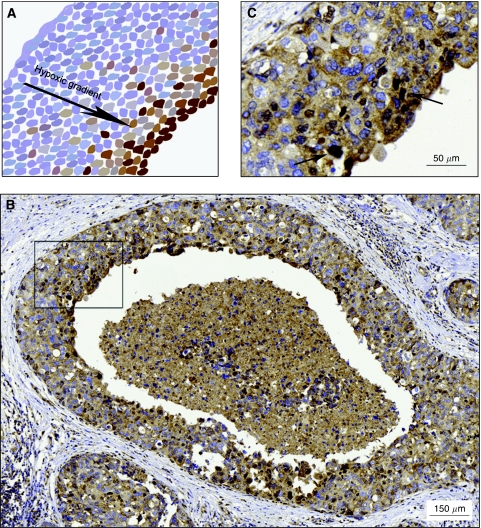
Immunohistochemical staining of Snail in DCIS. (**A**) A schematic representation of a typical HIF-1*α* staining appearance, with increasing staining intensity and number of positive cells approaching the central necrosis. (**B**) Nuclear expression of Snail was gradually increased in proximity to the central necrosis of DCIS. (**C**) Magnification ( × 20) of the framed area in panel **B**. Arrows indicate cells with intense nuclear Snail staining.

We continued by investigating the expression of Snail in a model system of hypoxia *in vivo*, non-invasive DCIS; DCIS lesions are often presented with areas of central necrosis where hypoxic tumour cells are surrounding the necrotic zone. Our aim was to elucidate whether these hypoxic cells displayed an EMT phenotype. Hypoxia-inducible factor-1*α* staining of full sections of DCIS confirmed the presence of hypoxia in cell layers adjacent to the central necrosis. Staining intensity and number of HIF-1*α*-positive cells however, varied noticeably between samples and lesions, suggesting antibody unreliability. The typical HIF-1*α* expression pattern that distinguishes DCIS is shown in Figure 6A (Helczynska *et al*, 2003). Cytoplasmic Snail expression was present in the majority of cells, whereas nuclear expression gradually increased approaching the necrosis, in a number of DCIS samples (Figure 6B and C). However, E-cadherin and vimentin expression was not changed in close proximity of the central necrosis (data not shown). Hence, DCIS did not display EMT-like features, but the results again confirmed that hypoxia induces Snail expression, and that this induction does not necessarily affect other EMT markers.

## Discussion

Epithelial–mesenchymal transition is described as a dynamic and reversible biological process. As cells from a solid tumour escape via the circulation and set metastases at new sites due to conversion of epithelial cells into mesenchymal cells, these cells are likely to revert back into epithelial cells at the distant site (Thiery and Sleeman, 2006; Peinado *et al*, 2007). We have shown in this study that breast cancer cells can exhibit partially induced EMT, where Snail induction as a result of hypoxia does not elicit a motile phenotype even though levels of mesenchymal markers such as vimentin are increased and E-cadherin levels decrease. Snail induction resulting from overexpression can conversely result in a migratory phenotype despite retention of vimentin and E-cadherin expression levels. Exposure of these breast cancer cell lines to severe hypoxia induced a shift towards an EMT phenotype, with elevated levels of Snail protein in all the cell lines, decreased expression of E-cadherin in MCF-7 cells and increased vimentin expression in MDA-MB-468 and MDA-MB-231 cells. However, increased cell migration that has previously been described for different cell lines as an effect of hypoxic exposure (Imai *et al*, 2003; Lester *et al*, 2007), was only observed for MDA-MB-468 cells. This suggests that these hypoxic breast cancer cells were only partially pushed towards EMT, with moderate increase in the E-cadherin repressor Snail. Accordingly, this partial induction is not sufficient to affect the migratory ability of these cell lines. Hypoxia is known to affect several factors involved in cell migration independently of EMT, and consequently the increased migratory capacity observed for hypoxic MDA-MB-468 cells did not seem to be an effect of increased Snail protein. The hypoxic response includes activation of numerous factors associated with cell motility and cytoskeletal structure (Semenza, 2003), and hence Snail-dependent migration is only one of several mechanism suggested to link hypoxia to an enhanced migratory propensity of tumour cells. The reduced migratory ability observed for MDA-MB-231 cells exposed to hypoxia may also be due to hypoxic effects.

Subcellular localisation of Snail has been reported to be regulated by interaction with GSK-3*β*, which predominantly resides in the cytoplasm (Zhou *et al*, 2004). Zhou *et al* (2004) demonstrated in MCF-7 cells that phosphorylation of Snail by GSK-3*β* occurs at two different consensus motifs: the first resulting in targeting Snail for ubiquitination and the second regulating its subcellular localisation. A Snail mutant exhibiting mutations in these phosphorylation motifs was shown to be much more stable and resided exclusively in the nucleus, suggesting that only Snail that is unable to interact with and become phosphorylated by GSK-3*β* is able to induce EMT in breast cancer cells. Interestingly, when screening for mutations in the two consensus motifs of Snail, no mutations could be identified, indicating that the upstream signalling is the crucial mechanism determining the function of Snail. Another protein that regulates the activity of Snail is the p21-activated kinase 1 (Pak1) (Yang *et al*, 2005). Pak1 phosphorylation of Snail promotes nuclear accumulation and transcription repression activity of the E-cadherin gene in breast cancer cells, and hence induction of EMT (Yang *et al*, 2005). However, further studies are required to delineate the mechanism underlying nuclear, cytoplasmic shuttling and subcellular localisation of Snail.

In the present study we demonstrated that subcellular localisation of Snail changes over time in breast cancer cell lines exposed to hypoxia. In MCF-7 cells E-cadherin was hardly detectable after 72 h, and expression of nuclear Snail was concurrently elevated, indicating that at this time point Snail was actively repressing E-cadherin-gene transcription in this cell line. Similarly, in MDA-MB-468 and MDA-MB-231 cells nuclear expression levels of Snail peaked at 96 h, around the same time that vimentin expression levels were markedly elevated in both cell lines. Nuclear expression of Snail did not increase as prominently in T-47D cells as in the other cell lines, which might explain the maintained levels of E-cadherin observed for this cell line. Our results indicate that hypoxia induces the expression of Snail, and after longer exposure Snail localises to the nucleus and exerts its EMT-inducing functions, resulting in downregulation of E-cadherin and upregulation of vimentin levels. The net result differs between cell lines, suggesting Snail to be more important as a regulator of EMT in some contexts, and may be inferior to other EMT regulators such as Slug or Twist in others.

The ‘EMT status’ of a cell line may not affect EMT regulation, that is, overexpression of Snail can induce a more migratory phenotype in both low-migratory, epithelial-like cell lines and in highly migratory, mesenchymal-like cell lines. The fact that Snail overexpression increased the migratory ability of both MCF-7 and T-47D cells, despite maintenance of E-cadherin expression in T-47D cells, suggests that E-cadherin expression does not exclude a migratory behaviour. The highly migratory MDA-MB-231 cells further increased their migratory capacity when overexpressing Snail, without exhibiting increased vimentin levels, further implying that Snail itself can drive migration independently of an EMT induction. Knocking down Snail resulted in significant reduction of the migratory capacity of MCF-7, MDA-MB-468 and MDA-MB-231 cells. The mechanism behind this shift in migration can again not be explained by regulation of E-cadherin or vimentin, since expression of neither of the two proteins was significantly changed by Snail silencing. Hence, another regulator of cell migration dependent or independent of EMT is possibly affected by Snail depletion. A plethora of factors play roles in the induction of EMT and regulating mechanisms generally differ between cell lines.

A number of studies describing Snail in breast cancer have been published, and the critical findings differ slightly among them. In one study Snail immunoreactivity was observed exclusively in the cytoplasm, contrasting to what is generally observed (Zhou *et al*, 2004), indicating antibody unreliability in this field of research. Becker *et al* (2007) reported 33% of breast cancers as being positive for nuclear Snail staining, similar to the 31% that we observed, using the same Snail antibody as in our study. Presence of nuclear Snail predicted a tumour phenotype displaying a more poorly differentiated histology and increased proliferation rate in premenopausal breast cancer, confirming the previously reported link between EMT and clinical aggressiveness (Cano *et al*, 2000; Blanco *et al*, 2002). Additionally, nuclear Snail protein was associated with more extensive hypoxia, defined by expression of the HIF-1*α*-inducible gene CAIX (Chaudary and Hill, 2006), implying a link between hypoxia and EMT in primary breast cancer. However, no correlation between Snail and HIF-1*α* expression was observed. Cytoplasmic Snail expression was not associated with HIF-1*α* or CAIX, or with any other clinicopathological variables, suggesting that expression of nuclear Snail plays the main role in determining the extent of clinical aggressiveness in breast cancer. A negative correlation between nuclear Snail and presence of ER*α* was observed, and this inverse relationship has previously been reported with a number of breast tumour materials analysed by microarray (Dhasarathy *et al*, 2007). Moreover, Snail has been reported to directly repress ER*α*-gene transcription in MCF-7 cells by binding to the promoter region (Dhasarathy *et al*, 2007). The ER–MTA3–E-cadherin pathway has emerged as a novel pathway in the regulation of ER signalling, and allelic alterations in these genes have been reported to be associated with both tumour-promoting and antitumour mechanisms (Cheng *et al*, 2001; Fearon, 2003). Furthermore, Snail suppresses the expression of the oestrogen-converting enzyme aromatase (Kumar, 2003), resulting in reduced levels of peripheral oestrogen converted from adipose tissue, which should be protective against breast cancer. The molecular function of Snail in breast epithelium is intricate and is further complicated by interaction with its downstream targets, such as the ER. Moreover, aetiological factors such as, for example, age at first full-term pregnancy, affects the importance of Snail as a susceptibility marker, and single-nucleotide polymorphism (SNP) of Snail has been shown to be of importance for the oestrogen-related risk factor in relation to breast cancer risk (Yu *et al*, 2006). In one study it was demonstrated that 17*β*-estradiol (E_2_) treatment, in an ER*α* mediated manner, resulted in increased metastatic potential, morphological changes characteristic of EMT and increased transcriptional activity of the Snail and Slug genes, accompanied by decreased levels of E-cadherin in an ovarian cancer cell line (Park *et al*, 2008). Silencing of either Snail or Slug caused reversion of the mesenchymal-like phenotype, and in addition treatment with the oestrogen antagonist ICI was shown to overcome the inhibitory effect of E_2_ on E-cadherin transcription, suggesting a crucial role for oestrogen and its receptor in EMT-dependent tumour progression. Interestingly, in the same study it was also demonstrated that presence of ER*β* had an opposing effect on ER*α*, in the context of EMT regulation, inhibiting the ER*α*-induced EMT (Park *et al*, 2008). These findings may explain the mechanism by which ER status regulates invasion and metastasis in breast cancer. Nevertheless, the effect of oestrogen and the role of ER in the context of Snail and EMT regulation remain elusive. The fact that patients with Snail-negative tumours exhibited impaired tamoxifen response, as compared with treated patients with Snail-positive tumours in our study, is intriguing, but the underlying mechanism is yet to be elucidated. Speculatively, Snail may affect the function of ER*α* in a way that potentiates the effect of the ER antagonist tamoxifen. In tumours with nuclear Snail expression, tamoxifen might exert its effect by inhibiting the function of Snail, as described previously for the antagonist ICI. This in turn may lead to a less invasive tumour phenotype, since EMT is reverted. In Snail-negative tumours tamoxifen would not be able to exert the same mechanistic effect, resulting in an abolished response. Expression of ER*β* may also influence the mechanism that renders patients with Snail-negative tumours non-responsive to tamoxifen. Evidently, further studies including large randomised clinical cohorts are needed to investigate the role of Snail as a treatment predictive marker in breast cancer.

Moreover, in DCIS cells adjacent to the central necrosis exhibited nuclear Snail, further confirming an association between hypoxia and EMT. However, expression of E-cadherin and vimentin was not changed in areas with nuclear Snail staining, suggesting that Snail can be upregulated without affecting the expression of other EMT markers *in vivo*, as was also demonstrated in our breast cancer cell lines.

Our present study provides further evidence of the critical role of Snail in tumorigenic behaviour of breast cancer cell lines, and its association with clinicopathological parameters defining a more aggressive tumour phenotype. We demonstrate that breast cancer cells can enter a hybrid state of EMT, which is distinguished by partial mesenchymal conversion, where some hallmarks of EMT are present and others absent. Loss of E-cadherin expression is a well-established definition of invasiveness (Mayer *et al*, 1993; Oka *et al*, 1993). It has, however, been debated whether this definition is reliable or not (Christiansen and Rajasekaran, 2006). The expression of E-cadherin was reported in one study to be present in the majority of invasive breast carcinomas examined (Hashizume *et al*, 1996). Furthermore, node-negative and node-positive tumours exhibited similar E-cadherin expression and no correlation between E-cadherin expression and metastatic status was observed. A number of additional reports show similar results regarding E-cadherin expression and tumour aggressiveness, implying that invasion is not necessarily dependent on a complete transition to a mesenchymal phenotype (Parker *et al*, 2001; Kovacs *et al*, 2003). Barrallo-Gimeno and co-workers argued that the primary function of Snail is to enhance cell movement, rather than to induce EMT (Barrallo-Gimeno and Nieto, 2005). In addition, Lou *et al* (2008) reported that the metastatic ability was not linked to the EMT status. A cell line with expression of vimentin and lack of E-cadherin expression was unable to metastasise, whereas another cell line exhibiting epithelial characteristics was capable of metastasising. Accordingly, the question if a primary tumour will metastasise or not may not be determined by the EMT phenotype of the tumour cells. The expression of Snail itself may hold more information about a tumour's invasive capacity and ability to metastasise than the actual EMT status.

## Figures and Tables

**Table 1:** Expression of EMT markers and migratory potential of breast cancer cell lines exposed to 0.1% oxygen

	**E-cadherin**	**Snail**	**Vimentin**	**Migration**
MCF-7	▾	▴	**—**	▪
T-47D	▪	▴	**—**	▪
MDA-MB-468	▪	▴	▴	▴
MDA-MB-231	**—**	▴	▴	▾

Abbreviations: EMT=epithelial–mesenchymal transition.

**—,** no expression; ▪, no change; ▾, downregulation; ▴, upregulation.

**Table 2:** Distribution of Snail staining category according to clinicopathological and molecular parameters of invasive breast cancer

	**Snail nuclear staining**		
**Variable**	**Negative (*n*=293)**	**Positive (*n*=91)**	***P*** a
*Tumour size (mm)*			0.901
⩽20	106	34	
>20	186	57	
Missing cases: 117			
			
*Tumour type*			0.361^b^
Ductal	246	75	
Lobular	20	7	
Medullary	14	7	
Missing cases: 131			
			
*Lymph node status*			0.893
Negative	80	26	
Positive	212	65	
Missing cases: 117			
			
*NHG*			0.003^b^
I	35	6	
II	132	30	
III	116	52	
Missing cases: 129			
			
*Ki67 positive* (%)			0.024^b^
0–1	41	7	
2–10	84	24	
11–25	67	27	
26–50	35	14	
51–100	31	15	
Missing cases: 155			
			
*ERα positive* (%)			0.002
<10	76	41	
⩾10	203	50	
Missing cases: 130			
			
*PR positive* (%)			0.006
<10	79	40	
⩾10	194	48	
Missing cases: 139			
			
*HIF-1α* (%)			0.129^b^
0–1	176	53	
2–10	36	18	
11–100	19	8	
Missing cases: 190			
			
*CAIX*			0.040
Negative	219	67	
Positive	21	14	
Missing cases: 179			

Abbreviations: CAIX=carbonic anhydrase-IX; ER=oestrogen receptor; HIF=hypoxia-inducible factor; NHG=Nottingham Histologic Grade; PR=progesterone receptor.

a Correlations were calculated using Fisher's exact test (two-sided) unless otherwise specified.

b Wilcoxon/Mann–Whitney test (two-sided).

**Table 3:** Multivariate Cox proportional-hazards model for Snail and treatment interaction based on ER*α*-positive breast cancer patients (*n*=325)^a^

		**Recurrence-free survival**		
**Variable**		**HR**	**95% CI**	***P***
Snail nuclear	Negative versus positive	0.252^b^	0.054–1.184	0.081
Treatment	Tamoxifen versus control	0.300^c^	0.116–0.776	0.013
Interaction variable^d^	Tamoxifen × Snail nuclear	2.911	1.009–8.395	0.048

Abbreviations: CI=confidence interval; ER=oestrogen receptor; HR=hazards ratio.

a Other factors included in the multivariate analysis were as follows: age (continuous), tumour grade (NHG I+II *vs* III), proliferation (Ki67 0–1, 2–10, 11–25, 26–50, 51–100%), nodal status (negative *vs* positive) and tumour size (⩽20 *vs* >20).

b Snail nuclear HR for the control group.

c Treatment HR for the Snail nuclear-positive group.

d Interaction variable states whether there is a difference in the treatment response in relation to Snail nuclear status.
