# Supplementary figures and images for: Hypoxia, Snail and incomplete epithelial–mesenchymal transition in breast cancer
Source: Br J Cancer. 2009 Oct 20;101(10):1769–81. doi: 10.1038/sj.bjc.6605369 (PMC2778529; doi:10.1038/sj.bjc.6605369)

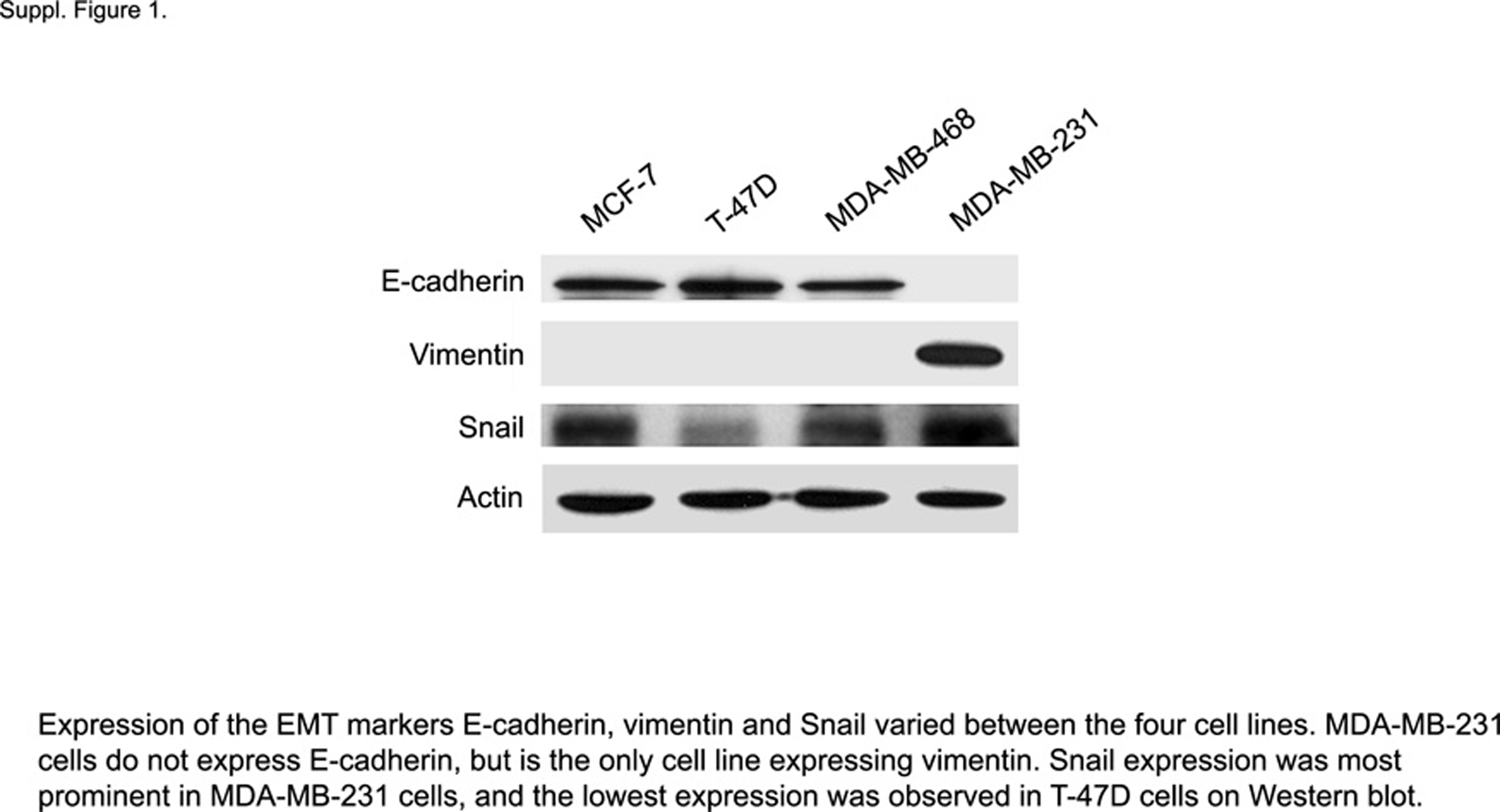

Supplement: Supplementary Figure 1 [file 6605369x1.tif]

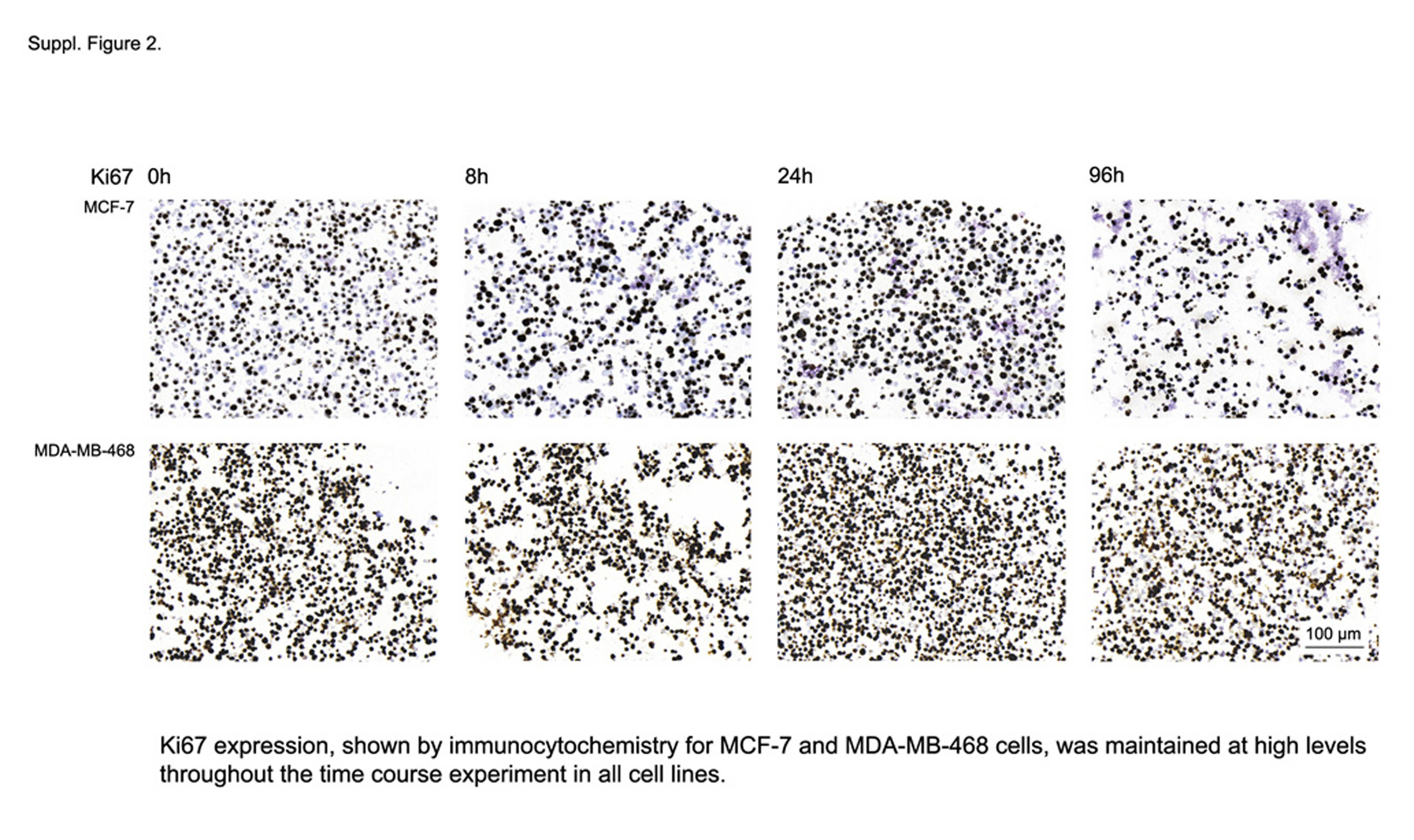

Supplement: Supplementary Figure 2 [file 6605369x2.tif]

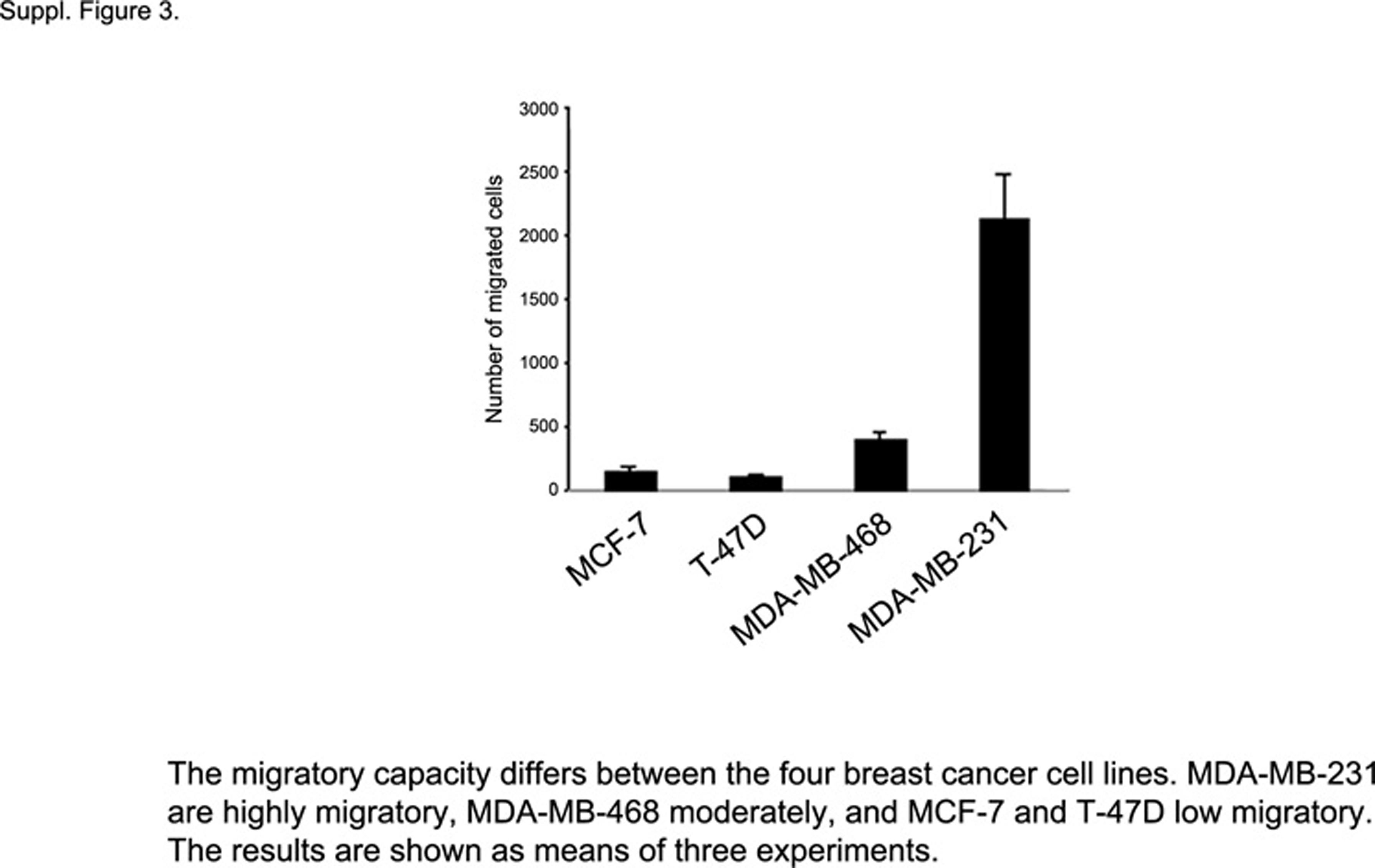

Supplement: Supplementary Figure 3 [file 6605369x3.tif]
